# Novel Anti-Viral Properties of the Herbal Extract of *Davallia mariesii* against Influenza A Virus

**DOI:** 10.3390/v16040523

**Published:** 2024-03-28

**Authors:** Yu-Li Chen, Pei-Yu Chao, Chung-Fan Hsieh, Pei-Wen Hsieh, Jim-Tong Horng

**Affiliations:** 1 Department of Biochemistry and Molecular Biology, College of Medicine, Chang Gung University, Taoyuan City 333, Taiwan; ylchen03@mail.cgust.edu.tw; 2 Research Center for Chinese Herbal Medicine, College of Human Ecology, Chang Gung University of Science and Technology, Taoyuan City 333, Taiwan; pewehs@mail.cgu.edu.tw; 3 Graduate Institute of Health Industry Technology, College of Human Ecology, Chang Gung University of Science and Technology, Taoyuan City 333, Taiwan; 4 Graduate Institute of Biomedical Sciences, College of Medicine, Chang Gung University, Taoyuan City 333, Taiwan; penny.chao@dupont.com; 5 Research Center for Emerging Viral Infections, College of Medicine, Chang Gung University, Taoyuan City 333, Taiwan; d000017560@cgu.edu.tw; 6 Department of Neurology, Linkou Chang Gung Memorial Hospital, Taoyuan City 333, Taiwan; 7 Graduate Institute of Natural Products, College of Medicine, Chang Gung University, Taoyuan City 333, Taiwan; 8 Molecular Infectious Disease Research Center, Chang Gung Memorial Hospital, Chang Gung University College of Medicine, Taoyuan City 333, Taiwan

**Keywords:** Gu-Sui-Bu, *Davallia mariesii*, hemagglutination, influenza A virus, neuraminidase

## Abstract

Gu-Sui-Bu, the dried rhizome of *Davallia mariesii*, is a traditional Chinese herbal remedy with a significant history of treating osteoporosis and inflammatory conditions. However, its potential as an anti-influenza agent and its underlying mechanisms of action remain unexplored. To obtain a more potent extract from *D. mariesii* and gain insights into its mechanism of action against influenza A virus (IAV), we utilized a partitioning process involving organic solvents and water, resulting in the isolation of butanolic subfractions of the *D. mariesii* extract (DMBE). DMBE exhibited a broad anti-viral spectrum, effectively inhibiting IAV, with an EC_50_ of 24.32 ± 6.19 µg/mL and a selectivity index of 6.05. We subsequently conducted a series of in vitro assays to evaluate the antiviral effects of DMBE and to uncover its mechanisms of action. DMBE was found to inhibit IAV during the early stages of infection by hindering the attachment of the virus onto and its penetration into host cells. Importantly, DMBE was observed to hinder IAV-mediated cell–cell fusion. It also inhibited neuraminidase activity, plaque size, and the expression levels of phospho-AKT. In summary, this study provides evidence for the effectiveness of *D. mariesii* as a complementary and alternative herbal remedy against IAV. Specifically, our data highlight DMBE’s capabilities in inhibiting viral entry and the release of virions.

## 1. Introduction

Influenza viruses, part of the Orthomyxoviridae family, are significant respiratory pathogens that caused influenza pandemics with global mortality and morbidity in 1918 (Spanish flu), 1957 (Asia flu), 1968 (Hong Kong flu), and 2009 (Swine flu) [1]. These viruses possess eight negative-sense single-strand RNA segments, encoding a minimum of 12 distinct viral proteins. These include hemagglutinin (HA), neuraminidase (NA), matrix proteins 1 (M1) and 2 (M2), polymerase acidic (PA), polymerase basic 1 (PB1), polymerase basic 2 (PB2), nucleoprotein (NP), and non-structural proteins 1 (NS1) and 2 (NS2) [2]. The influenza A virus (IAV) has the capability to infect both avian and mammalian species, whereas the influenza B virus is known for its ability to infect humans and seals. The distinct serotypes of IAV are identified based on two integral membrane glycoproteins, specifically hemagglutinin (spanning from HA1 to HA18) and neuraminidase (ranging from NA1 to NA11) [3]. Hemagglutinin, a major membrane glycoprotein on the IAV surface, plays a pivotal role in viral entry and is composed of HA1 and HA2. While HA1 is responsible for cell surface receptor binding, HA2 is involved in fusion and virus uncoating during the initial stage of infection [4].

During the initial phase of IAV infection, HA promotes viral entry by identifying and attaching to host cell surface receptors, such as glycans containing sialic acid [2]. This is followed by the virus’ entry into the cells, where it fuses with the endosomal membrane, and viral ribonucleoproteins (vRNPs) are transported into the cytoplasm and nucleus [5,6]. The newly synthesized vRNPs and progeny virions are then assembled near the cell membrane, priming them for bud formation. NA aids in the release of these progeny virions by cleaving the sialic acid of membrane glycoproteins [7]. Beyond facilitating the release and dissemination of progeny virions, the NA protein of IAV also performs the crucial functions of increasing virion infectivity and assisting in HA-mediated membrane fusion [8,9]. Besides its primary role, NA has the potential to enhance the expression of phospho-AKT through its interaction with plasma membrane proteins [10]. It was also observed that the upregulation of the cellular phosphatidylinositol 3-kinase (PI3K)/AKT signaling pathway is a prerequisite to viral activities. This underscores the possibility that the NA/AKT signaling axis could be explored as a new avenue for anti-influenza therapeutic interventions [11,12].

Oseltamivir and zanamivir, both sialic acid analogues, have been utilized successfully to curb influenza infection through their suppression of NA activity [13]. Despite their clinical effectiveness against IAV, the emergence of mutated strains featuring alterations in the NA catalytic site poses a significant global threat of future outbreaks [14]. Vaccines, which are always different, must be produced every year, and this process takes time. Furthermore, they are contraindicated in immunocompromised and immunosuppressed people.

Natural products serve as a pivotal cornerstone in the realm of drug development, primarily due to their rich diversity and abundance of chemical structures. As per recent data, the origins of almost 40% of drugs in clinical use can be traced back to natural products, highlighting their significance [15]. A salient example of this is the drug oseltamivir, which was formulated by leveraging a natural product, shikimic acid [16]. Moreover, polyphenols have been demonstrated to exhibit anti-IAV properties, both in vitro and in vivo, further underscoring the potential of natural products in pharmacological applications [17,18]. In our pursuit of a potent agent against IAV, we analyzed the potential of our comprehensive herb library. Our findings indicate that the aqueous extract of the *Davallia mariesii* rhizome offers protection against IAV-induced cell death. The *D. mariesii* rhizome has been utilized in traditional Chinese medicine, where it is known as “Gu-Sui-Bu”, to treat a variety of conditions, including osteoporosis, arteriosclerosis, and inflammation [19,20,21,22]. Alongside these uses, *D. mariesii* has also been proven to mitigate oxidative damage-induced apoptosis in neurons and enhance FcɛRI-mediated allergic responses in mast cells [20,23]. Polyphenols and flavonoids are frequently found secondary metabolites in *D. mariesii* [24,25]. However, to our knowledge, the antiviral properties of *D. mariesii* have not been previously examined [26,27]. In this study, we developed a protocol to produce an enhanced-activity version of *D. mariesii* butanolic extract (DMBE), and analyzed its mechanistic actions against IAV using several cell-based assays.

## 2. Materials and Methods

### 2.1. Cell Cultures and Viruses

Madin–Darby canine kidney (MDCK) cells were acquired from the American Type Culture Collection (ATCC; Manassas, VA, USA). MDCK cells were cultured in Dulbecco’s modified Eagle’s medium (DMEM; Invitrogen, Carlsbad, CA, USA) supplemented with 10% heat-inactivated fetal bovine serum (FBS; JRH Biosciences, Brooklyn, Victoria, Australia), 2 mM L-glutamine (Gibco, Gaithersburg, MD, USA), a mixture of 0.1 mM nonessential amino acids (NEAA) (Gibco), 100 U/mL penicillin, and 0.1 mg/mL streptomycin (Sigma-Aldrich, St. Louis, MO, USA). The influenza A virus A/WSN/33 (H1N1) was amplified in MDCK cells. Details on the origin and propagation of other influenza viruses, enteroviruses, herpes simplex virus (HSV), dengue virus, Japanese encephalitis virus, human coronavirus 229E, and adenovirus have been previously reported [28,29,30,31].

### 2.2. Preparation of Davallia Mariesii Butanolic Extract

The preparation of DMBE was conducted in accordance with a previously described protocol [32]. In brief, the process involved twice refluxing 20 g of raw *D. mariesii* material with 200 mL of ddH_2_O, each for a duration of 2 h. The resultant solutions were filtered and subsequently vacuum-concentrated to yield a crude water extract of *D. mariesii*. This extract (5.0 g) was dissolved in 200 mL of ddH_2_O and separated using ethyl acetate (EA) followed by *n*-butanol (BuOH), thus producing DMBE. For bioassay stock preparation, DMBE was initially dissolved in dimethyl sulfoxide (DMSO, 200 mg/mL), and then, centrifuged at 3000× *g* for 10 min. The supernatants were filtered using a 0.45 µm filter and stored at 4 °C for subsequent bioassays. Naringin, a major constituent of *D. mariesii*, was detected in the DMBE [23]. The high-performance liquid chromatography analysis results are illustrated in Figure S1. The voucher specimen of *D. mariesii* was archived in Chang Gung University’s herbarium (Taoyuan, Taiwan).

### 2.3. Cytotoxicity Assay

Cells were seeded at a density of 2 × 10^4^ cells per well within 96-well plates and allowed to rest overnight. Subsequently, they were exposed to varying concentrations of DMBE in E0. This medium, E0, is a blend of DMEM supplemented with 100 U/mL penicillin, 0.1 mg/mL streptomycin, 2 mM L-glutamine, and a 0.1 mM NEAA mixture. The incubation was carried out at a constant temperature of 37 °C for 72 h. After removing the medium, the cells underwent further incubation with a 3-(4,5-dimethylthiazol-2-yl)-2,5-diphenyltetrazolium bromide (MTT, Sigma-Aldrich) agent at 37 °C for 3 h. The absorbance at a wavelength of 570 nm for each well was assessed using an Lmax II384 ELISA reader (Molecular Devices, San José, CA, USA). The half-maximal cytotoxic concentration (CC_50_) of DMBE, defined as the concentration that resulted in 50% cell mortality, was determined as previously described [29]. 

### 2.4. Half-Maximal Effective Concentration (EC_50_) Assay

The EC_50_, or the concentration of DMBE required to inhibit 50% of the virus-induced cytopathic effect (CPE), was calculated using the Reed–Muench method [30]. Cell viability was assessed via two approaches: crystal violet staining and MTT reduction. MDCK cells were cultured in a 96-well plate, with each well holding 2 × 10^4^ cells. These cells were exposed to the influenza A virus (9 × TCID_50_, 50% tissue culture infective dose) and treated with either 0.1% DMSO or specific dosages of DMBE in E0.

After an incubation period of 72 h, the cells were fixed by adding 4% paraformaldehyde (PFA, Sigma-Aldrich) and allowing them to sit at room temperature for 1 h. The fixed cells were subsequently stained with 0.1% crystal violet for 30 min at room temperature. The optical density of the cells was measured using the Lmax II384 reader. For the MTT reduction method, MTT (0.5 mg/mL) was added to the cells, which were then incubated at a temperature of 37 °C for 3 h. The formazan crystals formed were dissolved by adding 150 mL/well of DMSO, and the optical density of the cells was evaluated at 570 nm (OD_570_) using the Lmax II384 reader [31,33].

### 2.5. Observation of Cytopathic Effect

MDCK cells, at a density of 5 × 10^5^ cells/well, were cultured in 6-well plates and infected with the influenza A virus WSN/33 strain at a multiplicity of infection (MOI) of 0.1 for a duration of 1 h at a temperature of 37 °C. Following infection, the cells were thoroughly rinsed twice with phosphate-buffered saline (PBS). Subsequently, they were incubated either with 0.1% DMSO or DMBE at a concentration of 20 µg/mL in E0 medium. Using a 20× objective lens on a Zeiss Axiovert 200 M microscope (Carl Zeiss, Göttingen, Germany), the CPE was meticulously documented [34].

### 2.6. Western Immunoblotting

MDCK cells, plated in 6-well dishes at a density of 5 × 10^5^ cells per well, were exposed to influenza A virus WSN/33 (MOI = 0.1) at 37 °C, concurrently with DMBE (20 µg/mL). Following a double rinse with PBS, the cells were collected at three distinct time points: 3, 6, and 9 hpi. The primary antibodies directed against PA (catalog number: GTX125932) and M1 (catalog number: B21) were sourced from GeneTex (Irvine, CA, USA) and ViroStat (Portland, ME, USA), respectively; tubulin (catalog number: GTX125932; GeneTex) served as an internal control. For AKT protein quantification, A549 cells in 6-well plates (5 × 10^5^ cells/well) were deprived of nutrients in E0 for 16 h. The cells were pre-chilled on ice for 1 h. The medium was removed, and the cells were treated with DMBE (20 µg/mL) and subsequently infected with the influenza A virus WSN/33 (MOI = 0.1) on ice for 10 min. The cell lysates were then gathered for an analysis of phosphorylated AKT and total AKT via western immunoblotting. 

Primary antibodies for AKT (Ser473, catalog number: #9272) and phosphorylated AKT (catalog number: #4060) were procured from Cell Signaling Technology (Danvers, MA, USA), whereas glyceraldehyde-3-phosphate dehydrogenase (GAPDH) was employed as the internal control (catalog number: GTX627408; GeneTex). Secondary antibodies—including anti-rabbit IgG (catalog number: GTX221666-01) and anti-goat IgG (catalog number: GTX228416-01)—were obtained from GeneTex. The amplification of signal detection was facilitated by an enhanced chemiluminescence Western blotting detection system (Millipore, Billerica, MA, USA) and assessed using a Fluro Chem HD2 (Alpha Innotech, Santa Clara, CA, USA) imaging system [29].

### 2.7. Time-of-Addition Assay

MDCK cells were initially seeded at a density of 5 × 10^5^ cells/well in six-well plates, following which they were infected with influenza A virus WSN/33 (MOI = 0.1) within the time interval of −1 to 0 h, lasting for 1 h (adsorption stage). The MDCK cells underwent incubation with DMBE at a concentration of 20 µg/mL at three principal time intervals, i.e., pre-adsorption (−3 to −1 h), adsorption (−1 to 0 h), and post-adsorption (0 to 9 h, 3 to 9 h, and 6 to 9 h). At 9 hpi, the culture supernatants were collected to determine the viral titer via a plaque formation assay.

### 2.8. Attachment and Penetration Assays

The attachment and penetration assays were carried out in accordance with a previously described methodology [35]. In the attachment assay, MDCK cells (2 × 10^4^ cells/well) were prepared in 96-well plates and pre-chilled on ice for 1 h, and the medium was removed. The cells were infected with the influenza A virus WSN/33 (3 × TCID_50_) in the presence of varied DMBE concentrations (3.13–100 µg/mL) at 4 °C for 2 h. Following this, any unattached viral particles were effectively removed by washing the cells twice with PBS. The treated MDCK cells were subsequently kept in E0, and after a 72 h incubation period, the cell viability maintained by DMBE was assessed through an MTT assay. In the penetration assay, the MDCK cells were chilled on ice for 20 min and the medium was removed. The cells were infected with influenza A virus WSN/33 (3 × TCID_50_) for 30 min. The medium was removed and the cells were washed twice with HBSS. Various concentrations of DMBE (3.13–100 µg/mL) in E0 were added and the cells were incubated at 37 °C for 1 h. Following this, the cells were subjected to brief exposure to HBSS at pH 2 for 1 min to inactivate any viruses that had not penetrated, before being neutralized with HBSS at pH 11. The cells were then incubated in E0 at 37 °C for 72 h. The effectiveness of DMBE in inhibiting viral penetration was then ascertained by measuring cell viability through an MTT assay.

### 2.9. Hemagglutination Inhibition Assay

The influenza A virus WSN/33 was prepared in a two-fold serial dilution and combined with a double volume of guinea pig red blood cells (RBCs) in a round-bottomed 96-well plate. Following a one-hour incubation period, the minimum virus titer triggering RBC aggregation was designated as 1 × HAv. In the hemagglutination inhibition assay, a 4 × HAv dose was employed. This involved pre-mixing the influenza A virus WSN/33 with DMBE at room temperature for 30 min. Afterward, RBCs in the 96-well plate were exposed to the virus/DMBE solution for an additional hour at room temperature. The final DMBE concentrations used in the assay varied between 0.24 and 125 µg/mL. The final percentage of RBCs in the solution was 0.5% [28].

### 2.10. Neuraminidase Inhibition Assay

The influenza A virus WSN/33 was combined with either 0.1% DMSO, DMBE (ranging from 6.5 to 200 µg/mL), or zanamivir (0.5 µM) in a buffer solution (33.3 mM MES, 4 mM CaCl_2_, at a pH of 6.5). This combination was maintained at a temperature of 37 °C for a duration of 30 min within a 96-well plate. Subsequently, the substrate 2′-(4-methylumbelliferyl)-α-D-N-acetylneuraminic acid (MU-NANA, Sigma-Aldrich) was integrated into the virus that had been previously treated with drug substances, with its final concentration being 50 µM. The treated virus was then incubated at 37 °C for 1 h. To inactivate the viral neuraminidase, a 0.1 M glycine buffer containing 25% alcohol was added. The neuraminidase enzymatic activities were assessed by measuring the fluorescence absorption at an excitation/emission wavelength of 360/460 nm using the Lmax II384 reader [29].

### 2.11. RNA Extraction and Quantitative Real-Time Polymerase Chain Reaction

MDCK cells were seeded at a density of 5 × 10^5^ cells/well in 6-well plates and infected with influenza A virus WSN/33 (MOI = 0.1) in the presence of DMBE (20 µg/mL) at a temperature of 37 °C for a duration of 1 h. Cell harvesting was performed at 0, 3, 6, and 9 hpi. Total intracellular RNA was then extracted using TRIzol reagent (Invitrogen). This RNA was used to prepare cDNA using M-MLV reverse transcriptase (Invitrogen). For the purpose of quantifying influenza A virus WSN/33 RNA levels through qRT-PCR, the following primer sequences were used: NP, 5′-AGC TGC ACA AAG AAC AAT GG-3′ (forward) and 5′-TGT GAG CAA CTG ACC CTC TC-3′ (reverse); M1, 5′-CGA GAT CGC ACA GAG ACT TG-3′ (forward) and 5′-GTG AGC GTG AAC ACA AAT CC-3′; GAPDH, 5′-AAG AAG GTG GTG AAG CAG CG-3′ (forward) and 5′-TCC ACC ACC CTG TTG CTG TA-3′ (reverse). GAPDH was employed as an internal control. The ratio of viral RNA to this internal control was then normalized against the control level at 0 hpi, which was defined as 1.0. All data were analyzed using the QuantStudio^TM^ Design and Analysis Software (version 1.4; Thermo Fisher Scientific, Waltham, MA, USA)

### 2.12. Plaque Reduction Assay

MDCK cells were seeded (5 × 10^5^ cells/well) in six-well plates and infected with influenza A virus WSN/33 (approximately 50 plaque-forming units) before being kept on ice for 1 h. The viral suspension was then removed and the cells were rinsed twice with PBS. These infected MDCK cells were subsequently exposed to E0 containing 0.3% agarose in the presence of either 0.1% DMSO, 0.5 µM zanavimir, or 2–100 µg/mL DMBE. Following a 72 h incubation period at 37 °C, the agarose was discarded and the cells were fixed with 4% PFA for 1 h at room temperature. This was followed by 20 min of staining with 0.1% crystal violet at room temperature. Finally, the plaque numbers and areas were analyzed using the MetaMorph offline imaging system software, version 7.1(Universal Imaging, West Chester, PA, USA).

### 2.13. Cell–Cell Fusion Assay

We adapted the cell–cell fusion assay to evaluate the HA’s membrane fusion and viral uncoating activity based on our previously published report [31]. We plated MDCK cells in 6-well plates at a density of 5 × 10^5^ cells per well. These cells were then infected with the influenza A virus WSN/33 (MOI = 0.1). Eight hours post-infection, we added trypsin (final concentration = 10 µg/mL) to the cells, aiming to stimulate hemagglutinin for a period of 30 min. This was immediately followed by a 30 min incubation with DMBE (20 µg/mL). After double washing with PBS, the cells were subjected to a 3 h incubation at 37 °C. Subsequently, they were fixed with 4% PFA for 16 h, which was then followed by staining with a 20% Giemsa stain (SG500, Sigma-Aldrich). Using the Lionheart FX imaging system (BioTek, Winooski, VT, USA), we observed the hemagglutinin-induced cell fusion. The syncytium was defined as having a minimum of five nuclei and was quantified by analyzing nine randomly selected views.

### 2.14. Data Analysis

Data were summarized as means ± standard deviations and analyzed using Student’s *t*-test. A two-tailed *p*-value of less than 0.05 was considered to indicate statistical significance.

## 3. Results

### 3.1. DMBE Exhibits a Wide-Ranging Inhibitory Effect against Different Viral Strains

In our search for effective antiviral agents, our scrutiny of an in-house-developed herbal library led us to discover that the water extract of *D. mariesii* exhibited a protective effect against cell death induced by the influenza A virus WSN/33.

**Figure 1:**
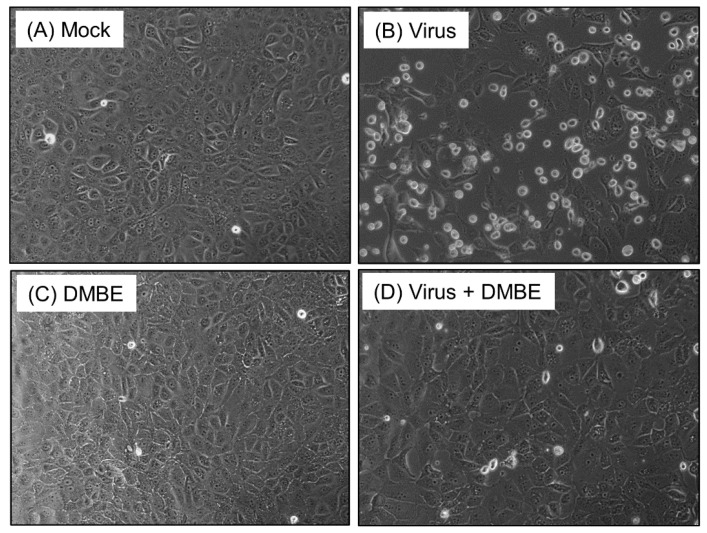
*D. mariesii* extract (DMBE) protects Madin-Darby canine kidney (MDCK) cells from virus-induced cytopathic effects. MDCK cells were infected with influenza A virus WSN/33 at a multiplicity of infection of 0.1, for a duration of 1 h, followed by a thorough rinse using phosphate-buffered saline (PBS). After washing, these cells were incubated under various conditions: 0.1% dimethyl sulfoxide (DMSO) (**A**), IAV infection (**B**), DMBE at a concentration of 20 µg/mL (**C**), or simultaneous IAV infection and DMBE exposure (**D**). After a period of 24 h, the morphological changes within the cells were observed and documented using a microscope equipped with a 20× objective lens.

Prior to investigating the underlying mechanisms, we fractionated the crude water extract of *D. mariesii* using organic solvents to help us obtain a more potent fraction. The crude extract of *D. mariesii* was dissolved in water and partitioned to yield EA, BuOH, and H_2_O fractions. The BuOH fraction (DMBE) demonstrated threefold inhibition of WSN/33-induced cell death in MDCK cells compared to the crude extract and the partition fractions. We assessed the cytotoxicity of DMBE in MDCK cells and calculated the selectivity index, obtaining a value of 6.05 (Table 1). The protective properties of DMBE against IAV were substantiated further by scrutinizing the morphological alterations in MDCK cells (Figure 1). When incubated with DMBE (20 µg/mL), the MDCK cells were effectively shielded from virus-induced cell death (Figure 1D). In contrast, the absence of DMBE led to a noticeable virus-induced cytopathic effect in MDCK cells, evidenced by the presence of detached, rounded cells (Figure 1B). We proceeded to explore the inhibitory range of DMBE in a variety of viral strains (Table 1). Our findings revealed that DMBE exhibits wide-ranging inhibition against different viral strain genera. Notably, the inhibition by DMBE was not restricted to the influenza A virus, but also extended to influenza B virus, enteroviruses (EV71 and EVD68), herpes simplex virus, adenovirus, dengue virus, Japanese encephalitis virus, and human coronavirus 229E (Table 1). Given that DMBE demonstrated potent anti-viral activity without inducing cytotoxicity in MDCK cells at a concentration of 20 µg/mL, we employed this dosage for subsequent mechanistic investigations.

### 3.2. DMBE Effectively Inhibits the Initial Stage of Influenza A Virus Infection

**Figure 2:**
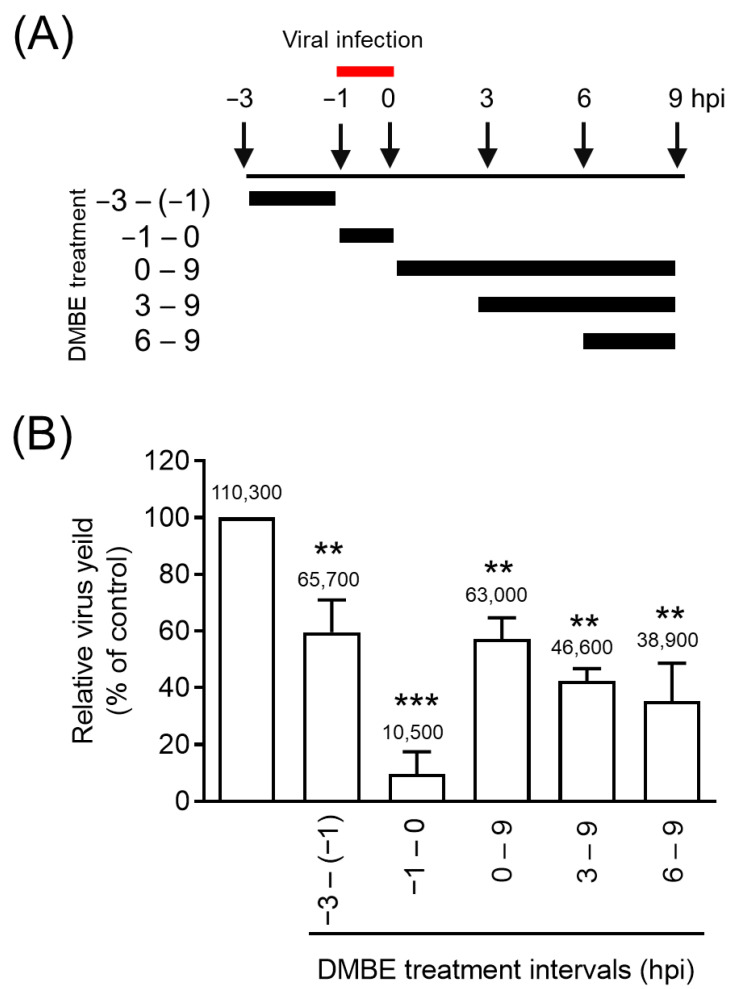
*D. mariesii* extract (DMBE) significantly reduces viral yield at the entry stage. (**A**) This diagram illustrates when DMBE treatment was administered in the time-of-addition assay. MDCK cells were exposed to DMBE (20 µg/mL) at various stages, i.e., before, during, or after the adsorption process of influenza A virus WSN/33 (multiplicity of infection, MOI = 0.1). (**B**) The harvested culture supernatants at the specified intervals were collected at 9 hpi. These samples were then used to determine the viral yield through a plaque assay. All experiments were conducted with biological replicates. The data, which are expressed as means ± standard deviations, were analyzed using two-tailed Student’s *t*-tests (*n* = 3). Statistical significance is indicated as ** *p* < 0.01 and *** *p* < 0.001, in comparison with the control group (0.1% dimethyl sulfoxide, DMSO). The mean values of plaque counts obtained from each experiment are represented above their respective bars.

Viral replication is a complex process encompassing five key steps: entry, transcription, translation, assembly, and budding. In our effort to understand the inhibitory mechanisms of DMBE against IAV infections, we carried out a time-of-addition assay (Figure 2). MDCK cells underwent infection via IAV adsorption, a process that took place from −1 to 0 hpi. Following this, the cells were subjected to incubation with DMBE at varying time intervals (Figure 2A). Throughout a single infection cycle, DMBE demonstrated prominent and significant inhibition against the strain A/WSN/33 of the influenza virus, particularly when cells were treated during the viral adsorption stage (−1 to 0 hpi; Figure 2B). Notably, only minimal inhibition occurred during the other DMBE treatment intervals (−3 to −1 hpi, 0 to 9 hpi, 3 to 9 hpi, and 6 to 9 hpi). These findings suggest that the primary target of DMBE is the initial stage of IAV infection.

### 3.3. DMBE Suppresses the Synthesis of Viral RNA and Proteins

**Figure 3:**
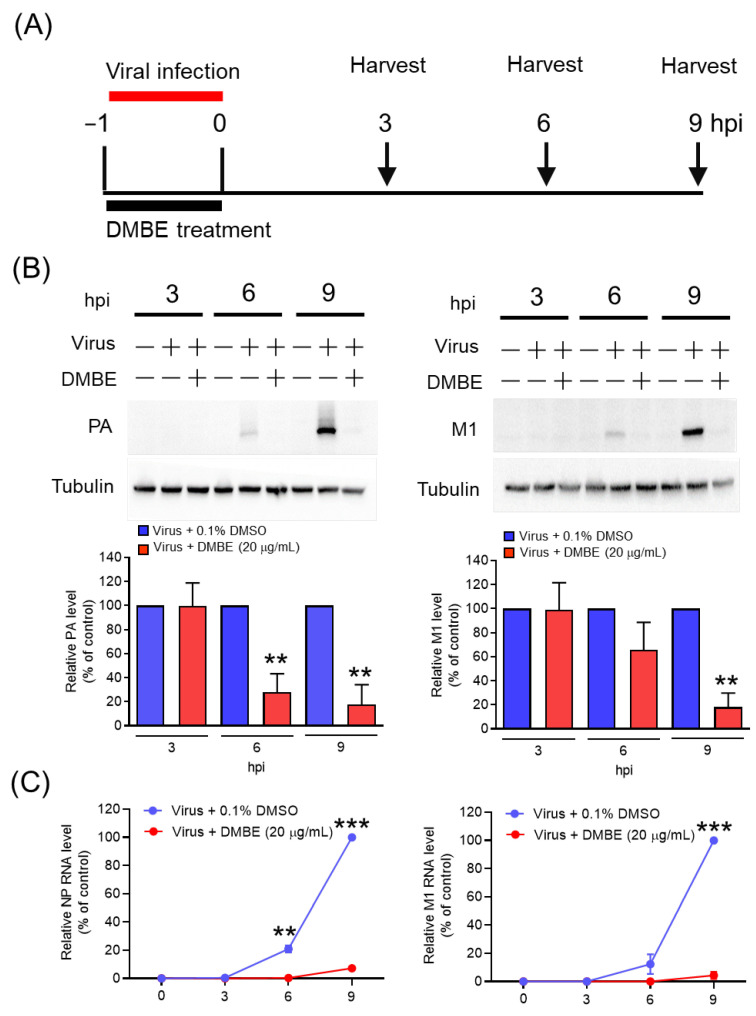
*D. mariesii* extract (DMBE) exerts inhibitory effects on viral RNA and protein synthesis. (**A**) Madin-Darby canine kidney (MDCK) cells were subjected to incubation with either 0.1% dimethyl sulfoxide (DMSO) or DMBE (20 µg/mL) during the course of influenza A virus WSN/33 infection (multiplicity of infection, MOI = 0.1). The cells were harvested at specified time intervals to evaluate the levels of intracellular viral protein and RNA. (**B**) The expression levels of polymerase acidic (PA) and matrix proteins 1 (M1) were detected and subsequently normalized with reference to tubulin. (**C**) The RNA levels of nucleoprotein (NP) and M1 were ascertained using qRT-PCR, with glyceraldehyde-3-phosphate dehydrogenase (GAPDH) serving as an internal control. All experiments were conducted with biological replicates. The data, which are expressed as means ± standard deviations, were analyzed using two-tailed Student’s *t*-tests (*n* = 3). Statistical significance is indicated as ** *p* < 0.01 and *** *p* < 0.001 in comparison with the control group (0.1% DMSO).

We proceeded to explore the impact of DMBE on viral RNA and protein synthesis (Figure 3). MDCK cells underwent infection with IAV (MOI = 0.1) in an environment containing either 0.1% DMSO or DMBE for a period of 1 h. Subsequent to the removal of unattached viruses, the cells were allowed to incubate and were harvested at the specified time intervals (3, 6, and 9 hpi) for viral RNA and protein analysis via immunoblotting and qRT-PCR, respectively (Figure 3A). The expression of viral proteins, as signified by PA and M1, as well as RNA (NP and M1), exhibited a time-dependent increment in the absence of DMBE treatment (Figure 3B). DMBE displayed a considerable ability to suppress not only the levels of viral proteins but also viral RNA expression (Figure 3B,C). Based on these findings, we infer that DMBE has the potential to curb viral entry, thereby resulting in the suppression of viral RNA and proteins and inhibiting viral replication.

### 3.4. DMBE Effectively Hampers the Attachment and Penetration of IAV

**Figure 4:**
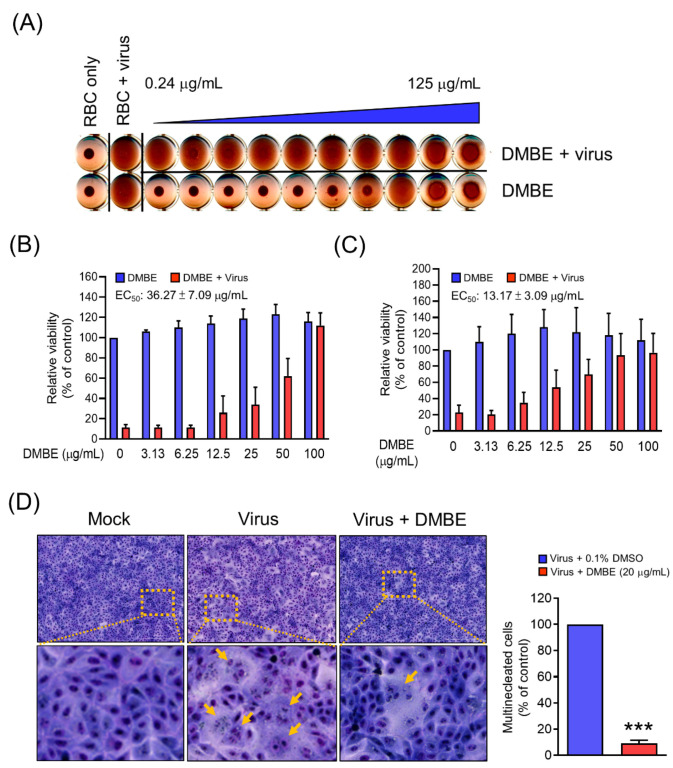
*D. mariesii* extract (DMBE) inhibits influenza virus A by impacting various stages of viral entry: hemagglutination (**A**), attachment (**B**), penetration (**C**), and hemagglutinin-mediated cell–cell fusion (**D**). In the hemagglutination stage (**A**), the influenza A virus was pre-incubated with either 0.1% dimethyl sulfoxide (DMSO) or DMBE for 30 min. This was followed by the addition of red blood cells into the virus/DMBE mixture, which was then incubated for an additional 60 min. DMBE doses were prepared with a two-fold dilution, ranging from right to left (0.24–125 µg/mL). During the attachment phase (**B**), an inhibition assay was performed. Madin-Darby canine kidney (MDCK) cells were pre-chilled on ice and simultaneously treated with DMBE and influenza A virus WSN/33 (3 × TCID_50_) for 1 h. After washing, the MDCK cells were cultured at 37 °C for 72 h. The cell viability, safeguarded by DMBE, was then evaluated by an MTT assay. For the penetration stage (**C**), a penetration inhibition assay was conducted. MDCK cells, once again pre-chilled, were initially infected by influenza A virus WSN/33 (9 × TCID_50_) for 30 min. After eliminating unattached virus through washing, the MDCK cells were incubated with either 0.1% DMSO or DMBE at 37 °C for 1 h. Acid HBSS was then added to the cells to deactivate any virus on the cell surface, followed by another washing stage. Cell viability was assessed after being culturing at 37 °C for 72 h. Finally, in the cell–cell fusion stage (**D**), MDCK cells were treated with trypsin for 30 min to activate hemagglutinin at 8 hpi. The cells were then incubated with DMBE (20 µg/mL) and cultured for 3 h. The extent of cell fusion was quantified by manually counting the multinucleated cells using a microscopic imaging system. All experiments were conducted with biological replicates. The data, which are expressed as means ± standard deviations, were analyzed using two-tailed Student’s *t*-tests (*n* = 3). Statistical significance is indicated as *** *p* < 0.001 in comparison with the control group (0.1% DMSO).

To elucidate the mechanistic function of DMBE in inhibiting IAV during the adsorption phase, we carried out a hemagglutinin inhibition assay. The primary objective was to investigate whether DMBE effectively hindered the binding activity between HA and its receptor (Figure 4A). The findings revealed that while IAV could instigate hemagglutination in RBCs, DMBE was unable to reverse this IAV-induced hemagglutination at concentrations up to 125 μg/mL. This implies that DMBE does not target the HA receptor binding region on HA1 (Figure 4A). Subsequently, we conducted a viral attachment assay to shed light on the impact of DMBE on host cells during viral entry. It was observed that DMBE effectively hindered IAV attachment, with an EC_50_ of 36.25 ± 7.09 µg/mL, an effect comparable to that indicated by the antivirus-induced CPE assay (Figure 4B versus Table 1). This points to the likelihood of DMBE targeting the HA receptor. Further investigations were conducted via a penetration assay and a cell–cell fusion assay to assess the influence of DMBE on endocytosis. In the penetration assay, the influenza virus was initially attached to the cells and subsequently incubated with or without DMBE during the penetration phase. The findings indicated that DMBE significantly impeded IAV penetration, with an EC_50_ of 13.17 ± 3.09 µg/mL (Figure 4C). DMBE’s inhibitory effect on endocytosis was further confirmed through a cell–cell fusion assay. Here, fusion was prominently reduced by more than 90%, with a DMBE concentration of 20 μg/mL (Figure 4D). In summary, these results suggest a strong likelihood that DMBE interrupts virus entry by impeding virus–cell membrane fusion during viral infection.

### 3.5. DMBE Inhibits Neuraminidase Activity and Prevents the Release of Progeny Viruses

**Figure 5:**
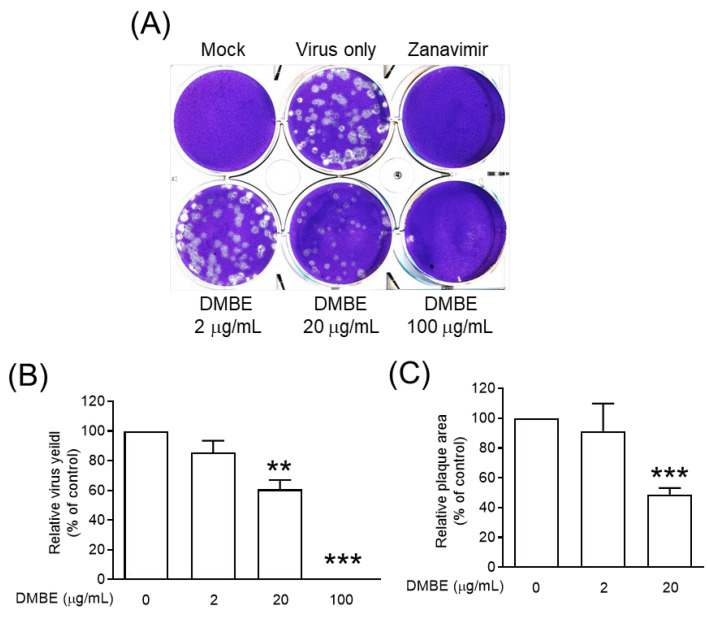
*D. mariesii* extract (DMBE) reduced plaque formation. (**A**–**C**) In plaque measurement assays, Madin-Darby canine kidney (MDCK) cells were infected with influenza A virus WSN/33 and subsequently overlaid with DMBE-infused agarose. The count and dimensions of the plaques were subsequently determined. All experiments were conducted with biological replicates. The data, which are expressed as means ± standard deviations, were analyzed using two-tailed Student’s *t*-tests (*n* = 3). Statistical significance is indicated as ** *p* < 0.01 and *** *p* < 0.001 in comparison with the control group (0.1% dimethyl sulfoxide, DMSO).

Neuraminidase, an essential glycoprotein on the surface of IAV, plays a key role in the egress of replicated viruses from infected cells. It achieves this by cleaving sialic acid, thereby enabling the release of newly formed virions [2]. Furthermore, research has linked neuraminidase to facilitating virus entry into cells [8,9]. We subsequently undertook an exploration into the effects of DMBE on both the enzymatic activity of NA and the release of progeny mediated by NA (Figure 5 and Figure S2). Our findings indicate that DMBE can inhibit IAV neuraminidase activity in a dose-dependent manner (Figure S2). Moreover, DMBE (20 µg/mL) was found to significantly and effectively reduce both the number and size of viral plaques (Figure 5A–C). These findings suggest that the decrease in IAV infection after DMBE treatment can be attributed, in part, to the modulation of NA activity.

### 3.6. The Efficacy of DMBE against the Influenza Virus Is Linked to the Downregulation of the PI3K/AKT Signaling Pathway

**Figure 6:**
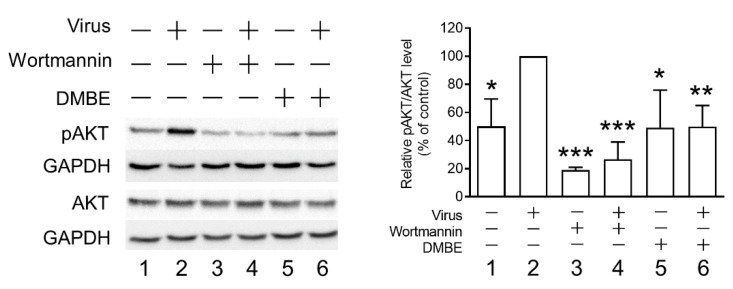
*D. mariesii* extract (DMBE) mitigates infection-induced AKT phosphorylation. A549 cells were initially chilled on ice for 1 h. The medium was removed, and the cells were treated with either 0.1% dimethyl sulfoxide (DMSO) or DMBE (at a concentration of 20 µg/mL) and subsequently infected with the influenza A virus WSN/33 (multiplicity of infection, MOI = 0.1) for 10 min on ice. The cell lysates were collected for quantifying the levels of pAKT using Western blot analysis. The densitometer-assisted quantification of pAKT expression is depicted on the right side of the figure. Wortmannin (100 nM) was employed as a positive control for comparison. All experiments were conducted with biological replicates. The data, which are expressed as means ± standard deviations, were analyzed using two-tailed Student’s *t*-tests (*n* = 3). Statistical significance is indicated as * *p* < 0.05, ** *p* < 0.01, and *** *p* < 0.001 in comparison with the control group (0.1% DMSO).

Prior research has established an association between kinase signaling pathways and the infectivity of the influenza virus [38]. Specifically, phosphorylated AKT (pAKT) was observed to increase following influenza virus infection. Furthermore, by blocking the PI3K/AKT signaling pathway, a decrease in viral entry was observed [39]. To further elucidate the mechanisms behind DMBE’s inhibition of IAV during viral entry, we measured pAKT levels in infected MDCK cells, both with and without DMBE treatment (Figure 6). The results of Western blotting revealed that DMBE was capable of reducing IAV-induced pAKT/AKT levels, a result comparable to that of wortmannin treatment (lane 2 versus lane 6 and lane 2 versus lane 4, Figure 6). Intriguingly, while wortmannin successfully suppressed the baseline levels of pAKT/AKT, DMBE failed to produce the same effect (lanes 3 and 5 versus lane 1, Figure 6), suggesting that DMBE may decrease pAKT/AKT expression through an indirect mechanism. This observation further bolsters the conclusion that DMBE exerts an inhibitory influence on IAV-mediated penetration and fusion assays (Figure 4).

## 4. Discussion

Influenza viruses have historically caused several outbreaks, marked by substantial morbidity and mortality. The emergence of drug-resistant strains intensifies the pressing demand for novel influenza treatments. Traditional medicine and natural products have routinely supplied a plethora of antiviral drugs [28,33,34,35,40]. Our current experimental findings indicate that DMBE can effectively curtail the cytopathic effect induced by IAV. A significantly greater protective impact of DMBE on cells in comparison with the untreated group was clearly noticeable (Figure 1). While we observed the cytotoxic effects of DMBE (CC_50_ = 147.18 ± 33.47 μg/mL), its selectivity index of 6.05 underscores its strong potency as a botanical anti-IAV agent (Table 1). This study specifically sought to delve into the anti-IAV effects of DMBE and shed light on its mechanism of action.

Initially, an experiment was carried out to explore the temporal dynamics of DMBE’s antiviral activity during the course of viral infection. The DMBE-treated group exhibited inhibitory effects consistently across all measured time intervals when compared to the untreated group, suggesting that DMBE is capable of cellular absorption and can thereby modulate the state of the cell. This interaction resulted in a noteworthy 50% reduction in viral infection (Figure 2). The most pronounced inhibitory effects were observed in the adsorption time span from −1 to 0 h, suggesting that DMBE, or its associated bioactive compound, can inhibit IAV by interfering with the virus’s entry into the cell.

Viral attachment, a critical event during the initial stage of virus infection, primarily relies on the co-regulation of HA and NA [8,9]. Our research indicates that DMBE can substantially hamper IAV’s attachment onto and penetration into MDCK cells (Figure 4). To delve deeper into DMBE’s role in virus attachment to host cells, we conducted a hemagglutination inhibition assay and a neuraminidase inhibition assay. Interestingly, DMBE showed no inhibitory effect on hemagglutination triggered by IAV HA, tested using RBCs from female guinea pigs (Figure 4A). Firstly, it is crucial to choose suitable RBC species for the HA inhibition assay, given the variations in the HA globular head’s affinity for α2,3/α2,6 sialic acid across disparate types and strains of influenza viruses. Future research could examine the impacts of varying RBC types, possibly using mammalian or human RBCs. Secondly, it is indeed plausible that DMBE may inactivate or kill the virus without diminishing the hemagglutination effect (Figure 4A), and the remaining viruses retain their infectious ability. Our current results indicate that DMBE primarily inhibits the early stage of IAV infection by reducing virus–cell membrane fusion. To specifically examine the inactivating/killing effects of DMBE, in a future study, we will first identify the antiviral components derived from DMBE through bioactivity-guided fractionation. Additionally, a centrifugal filtration inactivation assay, as described in our previous publication [35], will be performed to investigate whether DMBE or its components can directly inactivate/kill infectious viruses.

DMBE was found to inhibit neuraminidase activity, paving the way for subsequent cell-based experiments. Notably, DMBE not only effectively reduced the number of virus plaques, but also significantly curtailed their size (Figure 5). Interestingly, zanamivir, a specific NA inhibitor, results in noticeably smaller virus plaques. This contrasts with the roughly 40% inhibition effect that DMBE had on neuraminidase activity at a concentration of 100 µg/mL (Figure 5B). Furthermore, DMBE managed to entirely inhibit IAV plaque formation (Figure 5B,C). This implies that DMBE utilizes more complex mechanisms that go beyond simply inhibiting NA. Moreover, DMBE inhibited 90% of hemagglutinin-mediated cell–cell fusion at 20 µg/mL (Figure 4D) and 15% of neuraminidase activity at 25 µg/mL (Figure 5A). The observed alterations in the functions of HA and NA could potentially result in synergistic or additive effects during the initial stages of infection. This observation is consistent with findings from previous studies [8,9].

After cellular adhesion to the host cell, the event of clathrin-mediated endocytosis takes place. To determine DMBE’s capability to inhibit viral entry into cells, we performed penetration experiments. The results indicated that DMBE hinders viral entry, with an EC_50_ of approximately 13.17 ± 3.09 µg/mL (Figure 4C). The PI3K/AKT signaling pathway is known to facilitate influenza virus infection. Indeed, studies have shown that targeting AKT phosphorylation may provide an effective therapeutic strategy against IAV infections. By inhibiting AKT phosphorylation, the process of viral entry into cells can be effectively suppressed [39,41]. Furthermore, the interaction between NA and the plasma membrane protein carcinoembryonic antigen-related cell adhesion molecule 6 (CEACAM6 or C6) leads to the increased phosphorylation of AKT [10]. These data suggest that DMBE inhibits the NA-C6-AKT signaling axis, consequently preventing viral entry (Figure 5 and Figure 6).

IAV infection promotes the generation of reactive oxygen species, a process that can inflict substantial cell damage. However, antioxidants have the potential to temper the oxidative damage triggered by IAV [42,43]. A rich source of these beneficial antioxidants is found in *D. mariseii*, a plant known for its diversity in antioxidant phytoconstituents and potent radical neutralizing capabilities [19,23]. Intriguingly, *D*. *mariseii* not only exhibits antioxidative properties, but also demonstrates AKT-regulating abilities. This dual functionality provides a protective shield for neuronal cells against oxidative stress-induced cell death [23]. Therefore, it points to the potential for botanical applications that harness *D. mariseii*’s multifaceted capabilities.

The influenza A virus HA has two functions: it not only enables the virus to adhere to the host cell, but also facilitates membrane fusion during viral entry into the cell. This process primarily involves the fusion of the enveloping cell membrane with the virus’s own envelope, which then releases the viral RNP necessary for subsequent replication. However, when tested, DMBE showed no inhibitory effects on IAV-induced hemagglutination. Therefore, we designed a cell fusion experiment to further investigate DMBE’s potential impact on IAV HA. Our findings clearly demonstrated that DMBE effectively inhibits virus–cell membrane fusion (Figure 4D). Hence, there is a hypothesis that DMBE may influence the release of the virus RNP, potentially through HA2-mediated membrane fusion inhibition rather than affecting the HA1-mediated hemagglutination function (receptor binding) of HA. This deduction is drawn from findings suggesting that DMBE’s chemical components may potentially hinder the HA2 conformational change. Presently, efforts are being made to pinpoint the specific bioactive ingredient in DMBE responsible for this activity. The development of HA2 inhibitors has been a crucial approach in fighting IAV. Several HA2 fusion inhibitors, such as tert-butyl hydroquinone and stachyflin, are currently being investigated as promising antiviral treatments [44,45]. In addition, in a future study, MDBE-resistant virus selection and recombinant virus generation will be performed to provide direct evidence regarding the effects of DMBE on HA-mediated fusion.

The wide-ranging antiviral properties of certain agents can significantly enhance their effectiveness in drug development or environmental medication. It has been observed that DMBE exhibits a broad spectrum of antiviral activity (Table 1). In addition to inhibiting IAV, DMBE also suppresses the activity of other viruses such as influenza B virus, enteroviruses, HSV-1, dengue virus type 2, and the Japanese encephalitis virus. The vast antiviral spectrum of DMBE could be attributed to its multiple and diverse components. However, it is worth considering that a singular bioactive component within DMBE may have the capacity to target host factors, thus contributing to its extensive inhibition spectrum.

As per the Reaxys database, *Davallia* comprises 43 identified compounds, one being mangiferin. Published evidence has highlighted the potential anti-IAV properties of mangiferin, showcasing an IC_50_ of 0.82 ± 0.12 µM against NA activity [46]. To corroborate these findings, we conducted a cell-based assay to test the anti-IAV effectiveness of mangiferin. Regrettably, even at the maximum concentration of 100 µM, mangiferin failed to display any inhibitory effects against IAV infection (Figure S3). This leads us to surmise that mangiferin may not be the primary compound in DMBE accountable for inhibiting IAV.

Network pharmacology, which harnesses the potential of disease–target–drug interaction networks, has proven to be an effective tool in analyzing the underlying mechanisms of traditional Chinese medicines [47]. For instance, Xuanbai-Chengqi’s decoction’s protective qualities and mechanisms against lung and gut injuries in mice caused by IAV were uncovered through a comprehensive network pharmacology analysis [48]. Consequently, a synergistic network pharmacology approach that integrates the 43 compounds can be leveraged in future research to explore the correlation between the chemical composition of DMBE and its biological efficacy.

Interactions between viruses and lipids are pivotal for the replication of enveloped viruses, encompassing processes such as viral entry, assembly, and budding. Targeting these interactions represents a viable approach for creating broad-spectrum antiviral drugs. Recently, small molecules and peptides have demonstrated large-spectrum activity against various enveloped viruses, including SARS-CoV-2 and IAV. This activity involves compromising viral membrane integrity, inhibiting virus–cell membrane fusion, and interfering with cellular endocytosis [49,50,51]. In our study, the observed effects of DMBE on the enveloped virus and IAV-induced cell fusion suggested that it exhibits anti-lipid activity. To specifically examine the anti-lipid activity of DMBE, in a future study, the antiviral components derived from DMBE will be investigated. Additionally, experiments based on previous publications will be designed and performed to further explore this aspect.

## 5. Conclusions

This study pioneers the exploration of the antiviral properties of *D. mariesii*. Our findings demonstrate that *D. mariesii* effectively inhibits the entry of IAV by interfering with the functions of HA and NA, as well as by influencing the level of phosphorylated AKT. These insights substantiate the potential of *D. mariesii* as an effective herbal intervention against influenza virus-associated disorders.

## Figures and Tables

**Table 1:** Comparative analysis of the inhibition spectrum and cytotoxicity of *D. mariesii* extract (DMBE).

Cell Lines/Virus Strains	CC_50_^a^ (µg/mL)	EC_50_ (µg/mL)	Selectivity Index
Cytotoxic effects			
MDCK cells	147.18 ± 33.47	-	-
A549 cells	83.56 ± 9.51	-	-
RD cells	173.38 ± 9.19	-	-
BHK-21 cells	59.35 ± 22.19	-	-
Huh7 cells	>125	-	-
Influenza viruses			
A/WSN/33 (H1N1) ^c^	-	24.32 ± 6.19	6.05
B/TW/70233/05 ^c^	-	28.49 ± 13.84	5.17
B/TW/50344/19 ^b^	-	33.00 ± 3.93	4.46
Enteroviruses			
EVD68/TW/2795/14 ^c^	-	71.63 ± 3.38	2.42
HSV-1 ^b^	-	43.94 ± 3.25	1.90
Dengue virus type 2 ^c^	-	10.70 ± 1.38	5.55
Japanese encephalitis virus ^c^	-	15.76 ± 4.77	3.77
Human coronavirus 229E ^c^	-	44.00 ± 7.00	>2.84
Adenovirus ^b^	-	>200	-

^a^ Half-maximal cytotoxic concentration (CC_50_) was ascertained utilizing the diphenyltetrazolium bromide (MTT) assay method. ^b^ Half-maximal effective Concentration (EC_50_) was determined via a neutralization assay that incorporated crystal violet staining. ^c^ EC_50_ was ascertained through a neutralization assay with MTT used as the staining method. The influenza A virus (IAV) WSN/33 was procured from the ATCC and amplified in Madin-Darby canine kidney (MDCK) cells. The selectivity indexes of the clinical anti-IAV agents were as follows: oseltamivir (>1000) [36] and T-705 (>6000) [37].

## Data Availability

Data will be made available on request.

## Supplementary Materials

The following supporting information can be downloaded at: https://www.mdpi.com/article/10.3390/v16040523/s1, Figure S1: DMBE fingerprint. Figure S2: DMBE inhibits neuraminidase activity. Figure S3: Mangiferin does not impede cell death triggered by the influenza A virus.
