# Supplementary material for: Novel Anti-Viral Properties of the Herbal Extract of Davallia mariesii against Influenza A Virus
Source: Viruses. 2024 Mar 28;16(4):523. doi: 10.3390/v16040523 (PMC11054568; doi:10.3390/v16040523)
Supplement: Supplementary file 1 [file viruses-16-00523-s001.zip › viruses-2902373-supplementary.pdf]

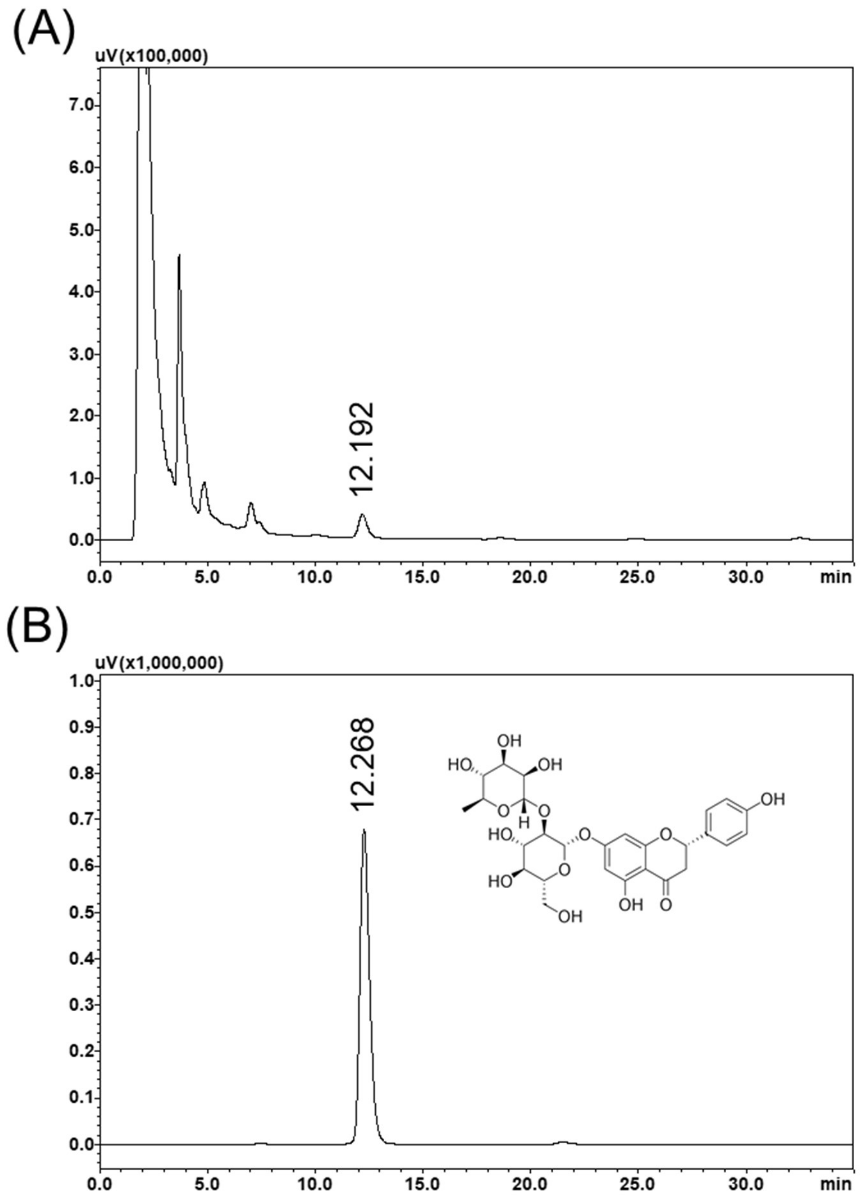

Figure S1. DMBE fingerprint. The analysis of DMBE and naringin was conducted using a Shimadzu Nexera-I LC-2040C-3D HPLC system (Kyoto, Japan) equipped with a UniverSilHS C18 HPLC column (250 × 4.6 mm; Fortis Technologies Ltd., Cheshiire, UK). Prior to analysis, DMBE (10 mg/mL) and naringin (0.5 μg/mL) were filtered through a 0.22-μm filter. The mobile phase comprised 40% methanol with 0.1% formic acid. The system was calibrated to a flow rate of 1.5 mL/min, while the detection wavelength and injection volume were set at 283 nm and 10 μL, respectively. The results showed that naringin was detected in DMBE at a retention time of 12.192 min (**A**). In contrast, naringin alone was identified at a slightly different retention time of 12.286 min (**B**).

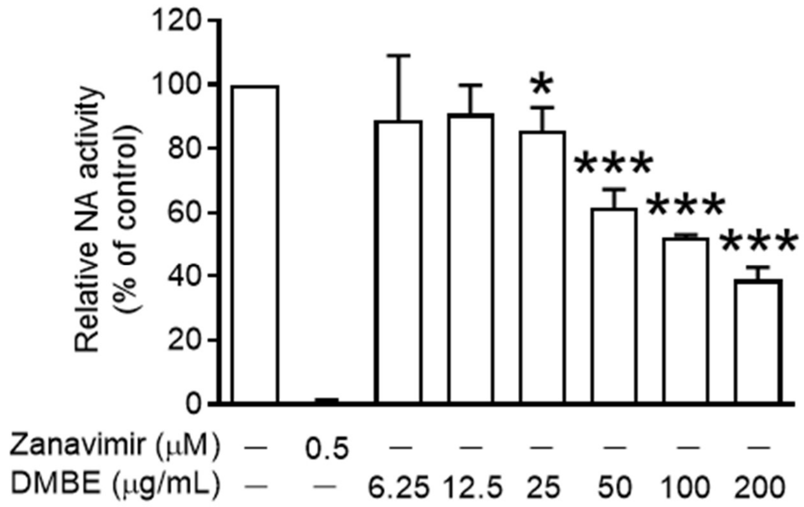

Figure S2. DMBE inhibits neuraminidase activity. Influenza A virus, WSN/33 was incubated with 0.1% DMSO or varying concentrations of DMBE. The degree of neuraminidase activity was assessed through the introduction of the MU-NANA substrate and subsequent measurement of fluorescence absorption at the excitation/emission wavelength of 355/460 nm. Zanamivir (0.5 μM) served as a control in this experiment. All experiments were conducted with biological replicates. The data, which are expressed as means ± standard deviations, were analyzed using two-tailed Student's *t*-tests ($n = 3$). Statistical significance is indicated as $^{*}p < 0.05$ and $^{***}p < 0.001$, in comparison with the control group (0.1% DMSO).

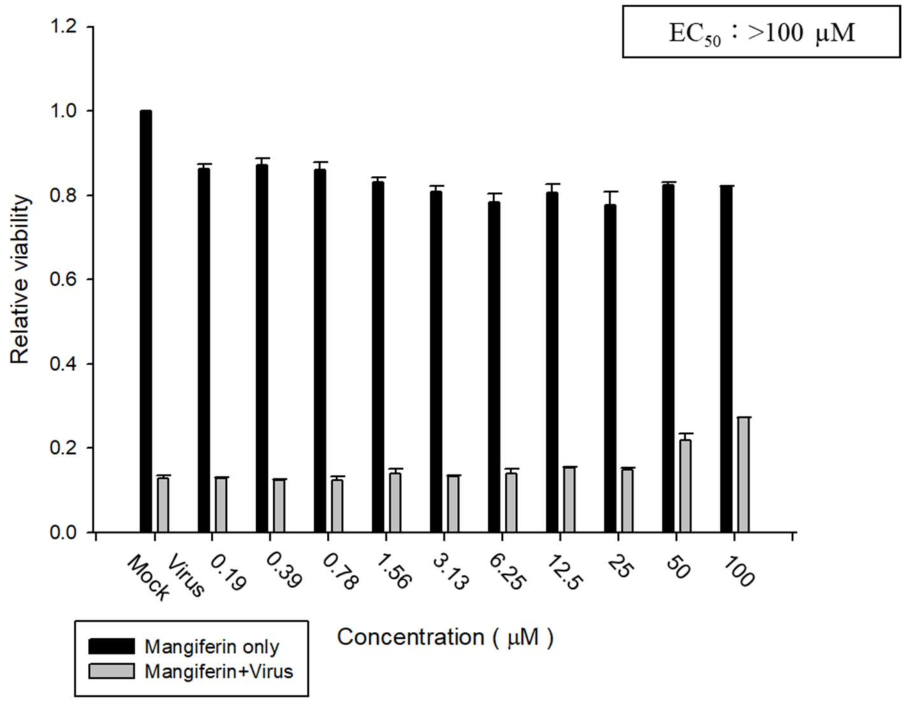

Figure S3. Mangiferin does not impede cell death triggered by the influenza A virus. MDCK cells, seeded in a 96-well plate at a density of 2×10^4 cells/well, were infected with the influenza A virus, WSN/33 ($9 \times TCID_{50}$). These cells were then treated with either 0.1% DMSO or specified doses of mangiferin in E0, and allowed to incubate for 72 h. Subsequently, MTT (0.5 mg/mL) was added to the cells and incubated at 37 °C for 3 h. To dissolve the resultant formazan crystals, dimethylsulfoxide (DMSO, 150 mL/well) was added. The optical density of the cells was then gauged at 570 nm (OD570) using a Lmax II384 reader.
